# µ-Opioid receptor antagonism facilitates the anxiolytic-like effect of oxytocin in mice

**DOI:** 10.1038/s41398-024-02830-1

**Published:** 2024-02-28

**Authors:** Khalin E. Nisbett, Leandro F. Vendruscolo, George F. Koob

**Affiliations:** 1 Graduate Program in Neuroscience, Graduate College, University of Illinois Chicago, Chicago, IL 60607 USA; 2 Stress & Addiction Neuroscience Unit, National Institute on Drug Abuse Intramural Research Program and National Institute on Alcohol Abuse and Alcoholism Division of Intramural Clinical and Biological Research, National Institutes of Health, Baltimore, MD 21224 USA; 3 Neurobiology of Addiction Section, Integrative Neuroscience Research Branch, National Institute on Drug Abuse Intramural Research Program, National Institutes of Health, Baltimore, MD 21224 USA

**Keywords:** Neuroscience, Drug discovery, Psychology

## Abstract

Mood and anxiety disorders are leading causes of disability worldwide and are major contributors to the global burden of diseases. Neuropeptides, such as oxytocin and opioid peptides, are important for emotion regulation. Previous studies have demonstrated that oxytocin reduced depression- and anxiety-like behavior in male and female mice, and opioid receptor activation reduced depression-like behavior. However, it remains unclear whether the endogenous opioid system interacts with the oxytocin system to facilitate emotion regulation in male and female mice. We hypothesized that opioid receptor blockade would inhibit the anxiolytic- and antidepressant-like effects of oxytocin. In this study, we systemically administered naloxone, a preferential μ−opioid receptor antagonist, and then intracerebroventricularly administered oxytocin. We then tested mice on the elevated zero maze and the tail suspension tests, respective tests of anxiety- and depression-like behavior. Contrary to our initial hypothesis, naloxone potentiated the anxiolytic-like, but not the antidepressant-like, effect of oxytocin. Using a selective μ−opioid receptor antagonist, D-Phe-Cys-Tyr-D-Trp-Arg-Thr-Pen-Thr-NH2, and a selective κ−opioid receptor antagonist, norbinaltorphimine, we demonstrate that μ−opioid receptor blockade potentiated the anxiolytic-like effect of oxytocin, whereas κ−opioid receptor blockade inhibited the oxytocin-induced anxiolytic-like effects. The present results suggest that endogenous opioids can regulate the oxytocin system to modulate anxiety-like behavior. Potential clinical implications of these findings are discussed.

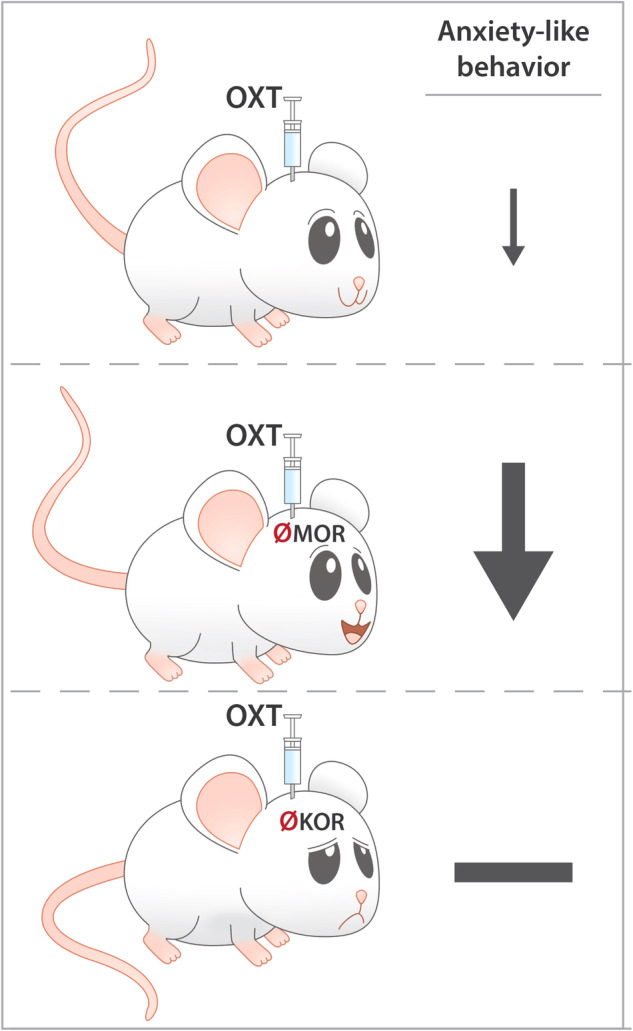

## Introduction

Mood and anxiety disorders are leading causes of disability worldwide, affecting approximately 18% of adults at least once in their lifetime [1]. The neurobiological substrates that contribute to emotion regulation are diverse, leaving significant gaps in our knowledge about how they interact and how they contribute to the development of and protection against depression and anxiety disorders. Additionally, many people do not respond positively to the United States Food and Drug Administration (FDA)-approved pharmacological treatments [2, 3]. Thus, new studies are needed to understand the mechanisms underlying these disorders to provide a broader spectrum of treatment options.

The central administration of oxytocin has been shown to improve preclinical stress-related measures, such as anxiety-like responses, depression-like responses, alcohol drinking, and stress-induced corticosterone levels [4–12]. Oxytocin, opioid peptides (e.g., β−endorphin and leu- and met-enkephalin) [13, 14], and their class A G-protein coupled oxytocin and μ−opioid receptors are key components of the neurocircuitry of the hypothalamus and the extended amygdala, i.e., brain regions that mediate stress and emotion regulation [15, 16].

Oxytocin release in the central nucleus of the amygdala has also been shown to gate fear responses in rodents [17–19], and systemic oxytocin administration has been shown to reduce alcohol drinking in a novel rodent model of comorbid post-traumatic stress disorder and alcohol use disorder [20]. In humans, studies have shown that intranasal oxytocin is effective at reducing subjective ratings related to social anxiety [21] and depression [22] when used as an adjunct to exposure therapy and escitalopram treatment, respectively.

Like oxytocin, preferential μ−opioid receptor agonists have demonstrated anxiolytic- and antidepressant-like properties in animal models [23–35]. For example, morphine and oxycodone reduced anxiety-like behavior in the elevated plus maze in stressed [23, 24] and stress-naïve rodents [25, 26]. Morphine, levorphanol, methadone, and tramadol produced antidepressant-like effects, i.e., decreased immobility, in the forced swim test [27–31]. Additionally, preclinical studies showed that an atypical antidepressant [36], tianeptine, which is a full μ−opioid receptor agonist, decreased depression- and anxiety-like behavior in rodents in the forced swim test and novelty-suppressed feeding test [32].

Previous studies demonstrated that μ−opioid receptor blockade inhibits oxytocin-mediated antinociception [37] and that μ−opioid [38] and oxytocin [39] receptors are expressed in brain regions relevant to anxiety- and depression-like behavior, and are simultaneously modulated during development [40]. Given these functional and morphological similarities between the opioid and oxytocin systems, we tested the hypothesis that the oxytocin and opioid systems interact to affect anxiety- and depression-like behavior. Specifically, we hypothesized that naloxone, a preferential μ−opioid receptor antagonist, would block the effect of oxytocin in tests of anxiety- and depression-like behavior in mice, and that the effect of naloxone would be mediated by μ−, but not κ−opioid receptors. To investigate these hypotheses, we administered naloxone (μ−opioid receptor-preferential opioid receptor antagonist) subcutaneously, D-Phe-Cys-Tyr-D-Trp-Arg-Thr-Pen-Thr-NH2 (CTAP; μ−opioid receptor-selective antagonist) intracerebroventricularly, or norbinaltorphimine (κ−opioid receptor selective antagonist) intraperitoneally prior to intracerebroventricular administration of oxytocin in male and female mice. Contrary to our initial hypothesis, we found that naloxone and CTAP potentiated the anxiolytic-like effect of oxytocin in the elevated zero maze, whereas norbinaltorphimine blocked this effect. Naloxone in combination with oxytocin did not reduce depression-like behavior (typical immobility), but naloxone reduced other measures (atypical immobility) in the tail suspension test.

## Materials & Methods

### Study approval

This study was conducted in accordance with the National Institutes of Health Guide for the Care and Use of Laboratory Animals and approved by the National Institute on Drug Abuse Intramural Research Program Animal Care and Use Committee.

### Animals

One hundred sixty male and 187 female C57Bl/6 J mice, 8-12 weeks old, weighing 18-30 g were used. Females were not ovariectomized because we learned from our previous study that oxytocin reduces anxiety-like behavior in naturally cycling females [12]. They were purchased from Jackson Laboratory (Bar Harbor, ME, USA) and acclimated to the laboratory for at least 1 week before the experiments began. The mice were housed in same-sex groups (2-4 per cage) in plastic cages (28 cm width × 17 cm length × 12 cm height) with free access to food and water except during testing procedures. The mice were kept in a room with a 12 h/12 h light/dark cycle (lights on at 7 AM) with controlled temperature (22 ± 2°C) and humidity (50-60%). All behavioral testing occurred during the light cycle because previous work in our laboratory has shown that this produces a response on the elevated zero maze where both anxiolytic-like and anxiogenic-like effects can be detected. This is consistent with our previous study that used a similar protocol [12]. The mice were randomly assigned to experimental groups after undergoing intracerebroventricular cannulation. The sample size was determined based on our previous publication to ensure adequate power to detect a pre-specified effect size [12].

### Intracerebroventricular cannulation

We conducted aseptic stereotaxic surgeries and drug administration procedures as previously described [12]. A 26-gauge, 5 mm, stainless steel guide cannula (Plastics One, Roanoke, VA, USA) was unilaterally implanted into the lateral ventricle using the following coordinates relative to bregma: −0.1 mm (anterior/posterior), −0.9 mm (medial/lateral), −1.6 mm (dorsal/ventral). The coordinates were based on *Paxinos and Franklin’s The Mouse Brain in Stereotaxic Coordinates* [41]. The cannula was fixed to the skull using skull screws and dental acrylic and closed using a stylet and an aluminum cap. Mice were then treated with meloxicam (5 mg/kg) postoperatively to reduce inflammation and placed in a warm cage (a heating blanket at 65-70°C was placed under the recovery cage) for at least 2 h before they were returned to their housing room. The mice were allowed to recover for at least 5 days before they were tested.

### Drug administration

Intracerebroventricular administration was performed as previously described [12]. After removal of the dust cap and dummy cannula, a 33-gauge infusion cannula that extended 1 mm beyond the guide cannula was inserted. The infusion cannula was attached to a polyethylene tube (PE-50; Plastics One, Roanoke, VA, USA) that was connected to a 10 μl Hamilton syringe driven by a microinfusion pump (KD Scientific, Holliston, MA, USA). Solutions were infused in a volume of 1-3 μl at a rate of 0.5 μl/min. The infusion cannula was left in place for at least 2 min to allow drug diffusion.

**Fig. 1:**
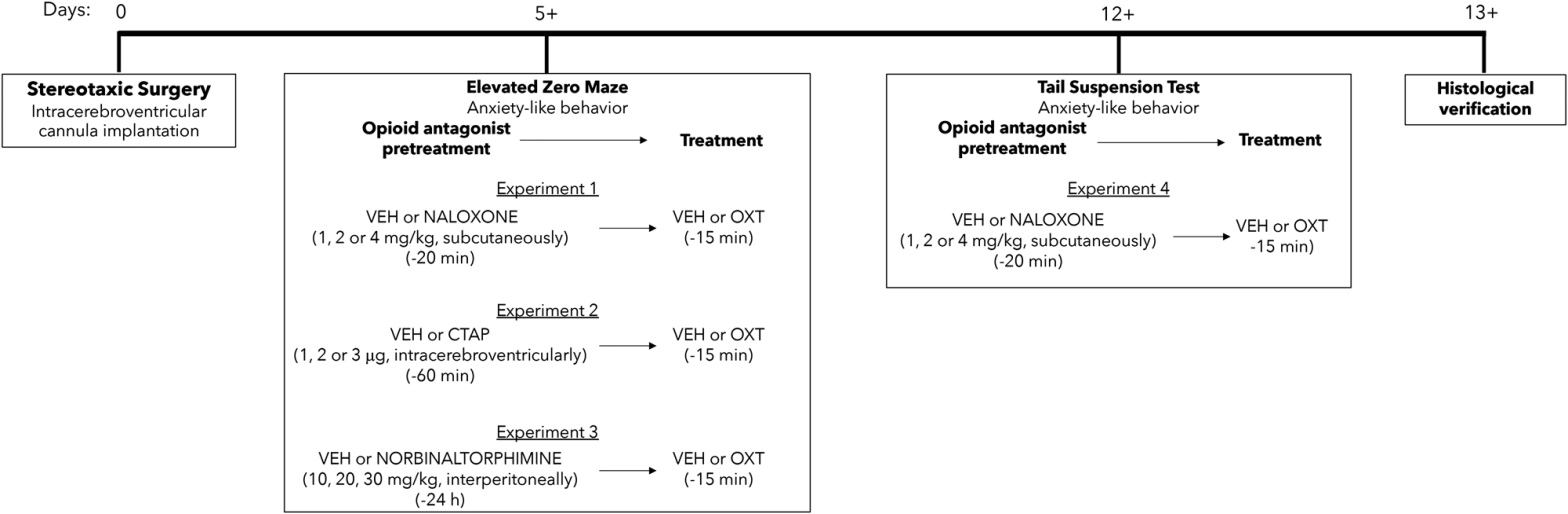
Experimental timeline. At least 5 days following intracerebroventricular cannula implantation, mice were tested on the elevated zero maze. Before testing, mice were pretreated with vehicle (VEH) or an opioid antagonist: naloxone (NLX), CTAP, or norbinaltorphimine (norBNI) and treated with vehicle (VEH) or oxytocin (OXT). At least 7 days after the elevated zero maze tests, mice previously exposed to vehicle or naloxone and vehicle or oxytocin were then tested on the tail suspension test. On the day of the experiment, mice were pretreated and treated with the same drug combination.

Vehicle (10 mL/kg saline) or naloxone (1, 2 or 4 mg/kg/10 mL) was administered subcutaneously 5 min prior to intracerebroventricular infusion of vehicle (saline in a 2 μL volume) or oxytocin (500 ng in a 2 μL volume) for Experiments 1 and 4. Vehicle (artificial cerebrospinal fluid in a 3 μL volume) or CTAP (1, 2 or 3 μg in a 1, 2 or 3 μL volume, respectively) was administered intracerebroventricularly 45 min prior to intracerebroventricular infusion of vehicle or oxytocin, as described above, for Experiment 2. Norbinaltorphimine is not selective for κ-opioid receptors until 4-24 h after administration [42]. Thus, vehicle (10 mL/kg saline) or norbinaltorphimine (10, 20, or 30 mg/kg/10 mL) was administered intraperitoneally 24 h prior to intracerebroventricular infusion of vehicle or oxytocin for Experiment 3. Mice were tested on the elevated zero maze or the tail suspension test 15 min after intracerebroventricular administration of oxytocin. Experimental drugs or controls were assigned to separate groups (i.e., a between-subjects design) of randomly selected mice (see Fig. 1).

### Elevated zero maze

The elevated zero maze test was conducted as previously reported [12]. Each mouse was allowed to explore the elevated zero maze for 5 min, and behavior was recorded using a wall-mounted Stoelting USB camera (Wood Dale, IL, USA). Behavior was analyzed in real-time by AnyMaze tracking software (Wood Dale, IL, USA). The percent time that the animal spent in the open quadrants (open zone occupancy) and number of entries into the open quadrants (open zone entries) were used as measures of anxiety-like behavior. Increases in open zone occupancy and entries were interpreted as a reduction of anxiety-like behavior.

### Tail suspension test

The tail suspension test was conducted as previously reported [12]. The 6 min test began immediately after the mouse was suspended. The test was recorded using a Stoelting USB camera that was mounted to a tripod and connected to a computer. The time that the animal spent immobile without trunk curling (typical immobility duration) and with trunk curling (atypical immobility duration) were measured and summed (total immobility duration). Total and typical immobility duration were used as measures of depression-like behavior. Atypical immobility duration was measured as a potential indicator of opioid-mediated mechanism of action [27]. All measures were scored by experimenters who were blind to experimental treatments.

### Histological verification of the injection site

After the final day of experimentation, the mice were euthanized by isoflurane overdose followed by cervical dislocation. Two microliters of Chicago Blue Dye solution (0.1% w/v) was microinfused through the guide cannulae. The brains were extracted and fresh frozen in isopentane on dry ice. Brains were sectioned at 50 μm until the cannula tracks and/or dye appeared. The location of the cannulae was identified using *Paxinos and Watson’s The Mouse Brain in Stereotaxic Coordinates* [43] for guidance. Brains that were accurately cannulated displayed a blue stain throughout the lateral, third, and fourth ventricles. Data from mice that were not successfully cannulated were excluded.

### Statistics

The statistical analyses were conducted using Prism software (GraphPad, San Diego, CA, USA). All data met the assumption of a normal distribution for statistical tests, and variance was similar between groups. The data were analyzed using two-way analyses of variance (ANOVAs). Analyses included sex (male *vs*. female) × opioid antagonist pretreatment (vehicle *vs.* 1-4 mg/kg naloxone, 1-3 μg/mouse CTAP, 10–30 mg/kg norbinaltorphimine) in oxytocin-treated mice, sex (male *vs*. female) × opioid antagonist pretreatment (vehicle *vs*. 4 mg/kg naloxone, 3 μg/mouse CTAP, 20 or 30 mg/kg norbinaltorphimine) in vehicle-treated mice, and sex (male *vs.* female) × treatment (vehicle *vs.* oxytocin) in vehicle-pretreated mice. ANOVAs that yielded a significant main effect of treatment or significant interaction effects were further analyzed using Holm-Sidak’s multiple-comparison *post hoc* test. Values of *p* < 0.05 were considered statistically significant for all tests. Data are expressed as means and standard errors of the means.

## Results

### Effect of opioid receptor antagonism on the anxiolytic-like effect of oxytocin in male and female mice using the elevated zero maze

**Fig. 2:**
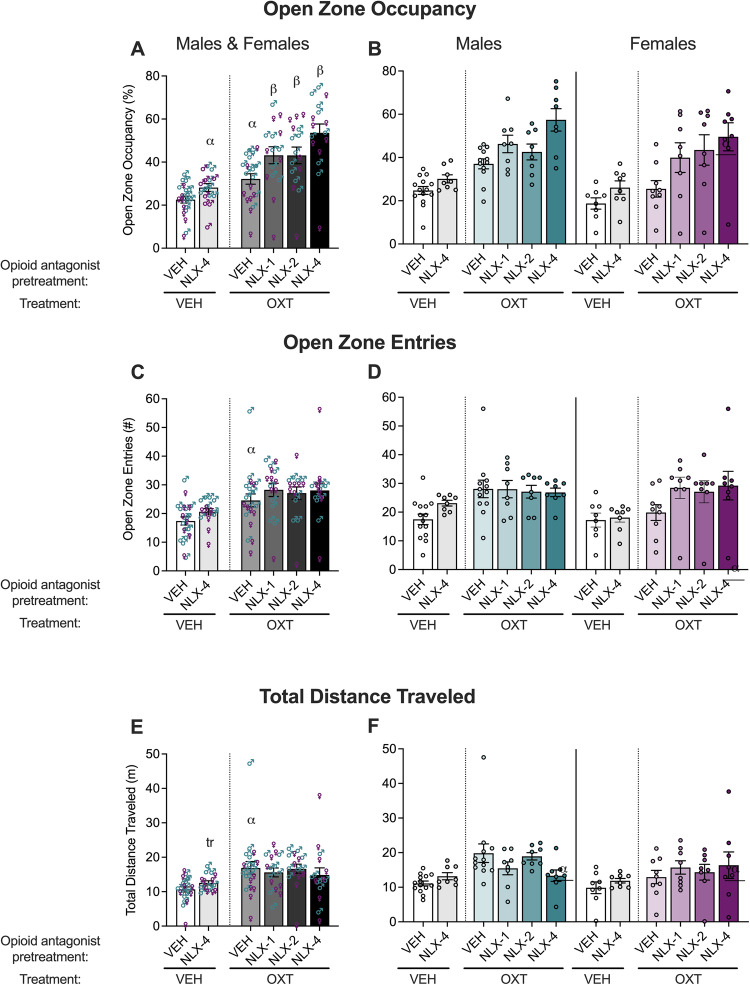
Anxiolytic-like effect of oxytocin is potentiated by naloxone in male and female mice in the elevated zero maze. Six experimental groups across both sexes were tested as VEH + VEH (*n*, males = 14, females = 8), NLX-4 + VEH (*n*, males = 8, females = 8), VEH + OXT (*n*, males = 12, females = 9), NLX-1 + OXT (*n*, males = 8, females = 8), NLX-2 + OXT (*n*, males = 8, females = 8) and NLX-4 + OXT (*n*, males = 8, females = 8). Oxytocin treatment (500 ng/mouse OXT; intracerebroventricular infusion) increased open zone occupancy (**A**), entries (**C**), and total distance traveled (**E**) compared with vehicle treatment (VEH; saline) in male and female vehicle-pretreated mice. Data are presented as means ± standard errors of the means. Oxytocin and naloxone pretreatment (1-4 mg/kg NLX; subcutaneous injection) further increased open zone occupancy (**A**), but not entries (**B**) or total distance traveled (**E**). Naloxone pretreatment (4 mg/kg) alone produced a significant increase in open zone occupancy (**A**) compared with vehicle pretreatment in vehicle-treated mice. Separate male and female data for open zone occupancy (**B**), entries (**D**), and total distance traveled (**F)** are also shown. ^α^*p* < 0.05, ^tr^*p* = 0.05, difference from VEH + VEH; ^β^*p* < 0.05, difference from VEH + OXT via two-way ANOVAs.

To test the interaction of naloxone with oxytocin, mice were subcutaneously pretreated with vehicle or naloxone then administered vehicle or oxytocin (500 ng/mouse) 5 min later via intracerebroventricular administration. The results for open zone occupancy, entries, and distance traveled in the elevated zero maze test are shown in Fig. 2.

The two-way ANOVA comparing all oxytocin-treated groups for open zone occupancy indicated a main effect of naloxone (naloxone > vehicle; *F*_3,61_ = 7.210, *p* = 0.0003) but no main effect of sex (*F*_1,61_ = 2.958, *p* = 0.0905), and no naloxone × sex interaction (*F*_3,61_ = 0.5784, *p* = 0.6314). *Post hoc* comparisons indicated that 1 mg/kg (*p* = 0.0337), 2 mg/kg (*p* = 0.0337), and 4 mg/kg (*p* < 0.0001) naloxone pretreatment, combined with oxytocin, produced higher open zone occupancy compared to vehicle pretreatment. The two-way ANOVAs comparing all oxytocin-treated groups for open zone entries and total distance traveled indicated no main effect of naloxone pretreatment (entries: *F*_3,61_ = 0.7975, *p* = 0.5000; distance: *F*_3,61_ = 0.2259, *p* = 0.8780), no main effect of sex (entries: *F*_1,61_ = 0.3222, *p* = 0.5724; distance: *F*_3,61_ = 1.465, *p* = 0.2309), and no naloxone × sex interaction (entries: *F*_3,61_ = 1.126, *p* = 0.3456; distance: *F*_3,61_ = 1.860, *p* = 0.3456).

The two-way ANOVA comparing vehicle *vs*. oxytocin alone in vehicle-pretreated mice on the elevated zero maze indicated a main effect of oxytocin (oxytocin > vehicle; *F*_1,39_ = 13.25, *p* = 0.0008) and a main effect of sex (male > female; *F*_1,39_ = 11.00, *p* = 0.0020), but no oxytocin × sex interaction (*F*_1,39_ = 1.097, *p* = 0.3014), for open zone occupancy. Similar analyses comparing vehicle *vs*. oxytocin alone for open zone entries indicated main effects of oxytocin (oxytocin > vehicle; *F*_1,39_ = 6.407, *p* = 0.0155) but no main effects of sex (*F*_1,39_ = 2.613, *p* = 0.1140) and no oxytocin × sex interaction (*F*_1,39_ = 2.313, *p* = 0.1364). The analysis of total distance traveled, indicated a main effect of oxytocin (oxytocin > vehicle; *F*_1,39_ = 9.530, *p* = 0.0037) and a main effect of sex (male > female; *F*_1,39_ = 4.517, *p* = 0.0400) but no oxytocin × sex interaction (*F*_1,39_ = 2.171, *p* = 0.1487).

The two-way ANOVA comparing vehicle *vs*. 4 mg/kg-naloxone pretreatment in vehicle-treated mice indicated a main effect of naloxone on open zone occupancy (naloxone > vehicle; *F*_1,34_ = 7.166, *p* = 0.0114), but not open zone entries (*F*_1,34_ = 2.826, *p* = 0.1019) and a trend toward significance for total distance traveled (*F*_1,34_ = 3.937, *p* = 0.0554). A main effect of sex was indicated for open zone occupancy (males > females; *F*_1,34_ = 4.303, *p* = 0.0457), but not for open zone entries (*F*_1,34_ = 1.843, *p* = 0.1835) nor total distance traveled (*F*_1,34_ = 1.499, *p* = 0.2293). These analyses did not show a naloxone × sex interaction for open zone occupancy (*F*_1,34_ = 0.1801, *p* = 0.6740), entries (*F*_1,34_ = 1.509, *p* = 0.2277), or total distance traveled (*F*_1,34_ = 0.0005886, *p* = 0.9808).

Altogether, the present results replicate our previous study showing that oxytocin is anxiolytic-like in male and female mice [12] and further suggest that blocking μ−opioid receptors can potentiate this effect.

### Effect of μ−opioid receptor antagonism on the anxiolytic-like effect of oxytocin in male and female mice using the elevated zero maze

**Fig. 3:**
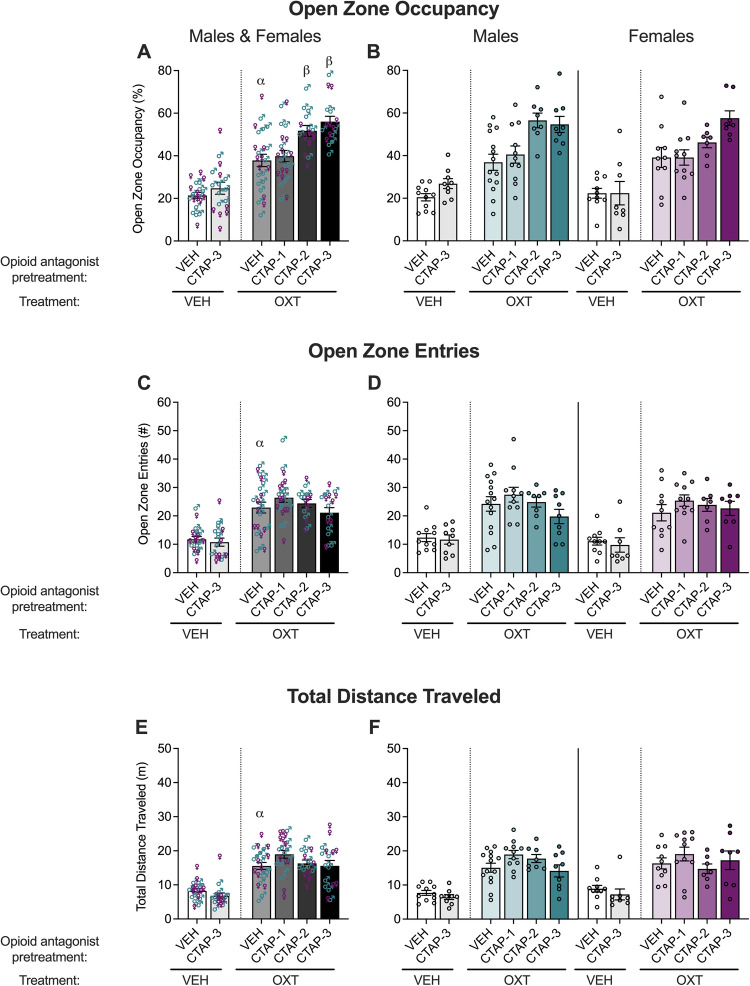
Anxiolytic-like effect of oxytocin is potentiated by CTAP in male and female mice in the elevated zero maze. Six experimental groups were tested as VEH + VEH (*n*, males = 11, females = 10), VEH + CTAP-3 (*n*, males = 9, females = 8), VEH + OXT (*n*, males = 14, females = 10), CTAP-1 + OXT (*n*, males = 11, females = 11), CTAP-2 + OXT (*n*, males = 8, females = 7) and CTAP-3 + OXT (*n*, males = 9, females = 8). Open zone occupancy (**A**), entries (**C**), and total distance traveled (**E**) were increased by oxytocin treatment (500 ng/mouse OXT; intracerebroventricular infusion) compared with vehicle treatment (VEH; saline) in male and female vehicle-pretreated mice. Data are presented as means ± standard errors of the means. CTAP pretreatment (1-3 μg/mouse; intracerebroventricular infusion) potentiated the effect of oxytocin on open zone occupancy (**A**) but not open zone entries (**C**), or total distance traveled (**E**). CTAP pretreatment had no effect on open zone occupancy (**A**), open zone entries (**C**) nor total distance traveled (**E**) compared with vehicle pretreatment in vehicle-treated mice. Separate male and female data for open zone occupancy (**B)**, entries (**D**), and total distance traveled (**F**) are also shown. ^α^*p* < 0.05, difference from VEH + VEH; ^β^*p* < 0.05, difference from VEH + OXT, via two-way ANOVAs.

The selective μ−opioid receptor antagonist, CTAP, was tested to determine whether the effect of naloxone was mediated by μ−opioid receptors. Vehicle or CTAP (1-3 μg/mouse; intracerebroventricular infusion) was administered 45 min prior to oxytocin. The results for open zone occupancy, entries, and total distance traveled in the elevated zero maze test are shown in Fig. 3.

Two-way ANOVAs indicated a main effect of CTAP pretreatment for open zone occupancy (CTAP > vehicle; *F*_3,70_ = 9.990, *p* < 0.0001), but not for open zone entries (*F*_3,70_ = 1.613, *p* = 0.1940) or total distance traveled (*F*_3,70_ = 1.896, *p* = 0.1382). There were no main effects of sex (occupancy: *F*_1,70_ = 0.3252, *p* = 0.5703; entries: *F*_1,70_ = 0.2151, *p* = 0.6442; distance: *F*_1,70_ = 0.06746, *p* = 0.7958) and no CTAP × sex interaction (occupancy: *F*_3,70_ = 1.1021, *p* = 0.3885; entries: *F*_3,70_ = 0.5129, *p* = 0.6747; distance: *F*_3,70_ = 0.9439, *p* = 0.4246) for either measure. *Post hoc* tests indicated that 2 μg (*p* = 0.0043) and 3 μg (*p* < 0.0001) but not 1 μg (*p* = 0.9437) CTAP potentiated the anxiolytic-like effect of oxytocin.

The two-way ANOVA comparing vehicle and oxytocin treatment in vehicle-pretreated mice on open zone occupancy indicated a main effect of oxytocin (oxytocin > vehicle; occupancy: *F*_1,41_ = 23.23, *p* < 0.0001; entries: *F*_1,41_ = 23.28, *p* < 0.0001, distance: *F*_1,41_ = 35.68, *p* < 0.0001), no main effect of sex (occupancy: *F*_1,41_ = 0.3719, *p* = 0.5454; entries: *F*_1,41_ = 0.9345, *p* = 0.3394; distance: *F*_1,41_ = 0.9221, *p* = 0.3426), and no oxytocin × sex interaction effect (occupancy: *F*_1,41_ = 0.003402, *p* = 0.9538; entries: *F*_1,41_ = 0.1670, *p* = 0.6849; distance: *F*_1,41_ = 0.0004560, *p* = 0.9831) for the reported measures.

Two-way ANOVAs computed to analyze vehicle *vs*. 3 μg/mouse CTAP on open zone occupancy in vehicle-treated mice did not demonstrate a main effect of CTAP treatment (occupancy: *F*_1,34_ = 1.143, *p* = 0.2925; entries: *F*_1,34_ = 0.03632, *p* = 0.8500; distance: *F*_1,34_ = 2.083, *p* = 0.1581) or sex (*F*_1,34_ = 0.1795, *p* = 0.6745; entries: *F*_1,34_ = 0.8614, *p* = 0.3599; distance: *F*_1,34_ = 0.8647, *p* = 0.3590) or a CTAP-3 × sex interaction (occupancy: *F*_1,34_ = 1.113, *p* = 0.2988, entries: *F*_1,34_ = 0.03632, *p* = 0.8500; distance: *F*_1,34_ = 0.05011, *p* = 0.8242) for the reported measures.

### Effect of κ−opioid receptor antagonism on the anxiolytic-like effect of oxytocin in male and female mice using the elevated zero maze

**Fig. 4:**
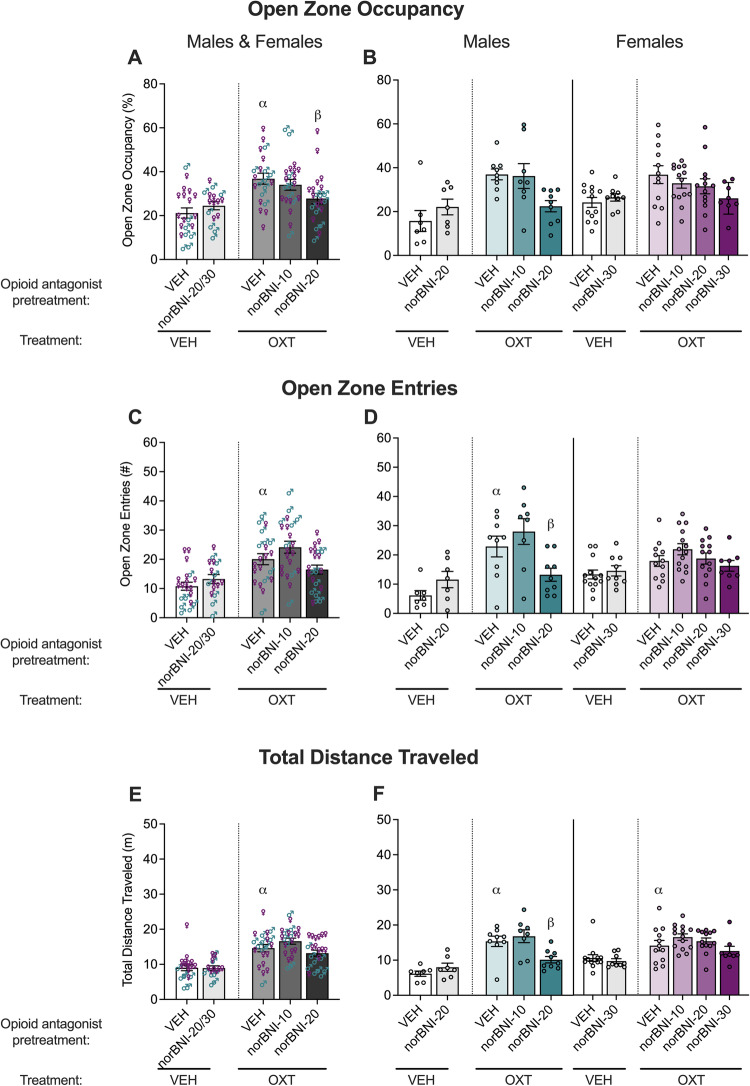
Anxiolytic-like effect of oxytocin is reduced by norbinaltorphimine in male and female mice in the elevated zero maze. The male experimental groups were VEH + VEH (*n* = 7), norBNI-20 + VEH (*n* = 7), VEH + OXT (*n* = 9), norBNI-10 + OXT (*n* = 8) and norBNI-20 + OXT (*n* = 9), and the females experimental groups were VEH + VEH (*n* = 13), norBNI-30 + VEH (*n* = 9), VEH + OXT (*n* = 12), norBNI-10 + OXT (*n* = 14), norBNI-20 + OXT (*n* = 13), and norBNI-30 + OXT (*n* = 8). Oxytocin treatment (500 ng/mouse; intracerebroventricular infusion) increased open zone occupancy (**A**), entries (**C**, **D**), and total distance traveled (**E**, **F**) compared with vehicle (VEH; saline) in male and female vehicle-pretreated mice in the elevated zero maze. Data are presented as means ± standard errors of the means. Norbinaltorphimine pretreatment (10–30 mg/kg norBNI; intraperitoneal injection) blocked the effect of oxytocin on open zone occupancy (**A**), open zone entries (**C**, **D**), and total distance traveled (**E**, **F**), but norBNI alone did change either measure compared with vehicle pretreatment in vehicle-treated mice. Separate male and female data for open zone occupancy (**B**), entries (**D**), and total distance traveled (**F**) are also shown. ^α^*p* < 0.05, difference from VEH + VEH; ^β^*p* < 0.05, difference from VEH + OXT, via two-way ANOVAs.

Previous studies suggest that like μ−opioid receptor agonists, κ−opioid receptor agonists can delay oxytocin-mediated function and inhibit oxytocin release from the neurohypophysis, whereas κ−opioid receptor antagonists, like μ−opioid receptor antagonists, had the opposite effect [44–46]. Thus, we determined whether κ−opioid receptor blockade contributed to the initial potentiating effect of naloxone by administering vehicle or norbinaltorphimine 24 h before vehicle or oxytocin. Males were tested with two doses of norbinaltorphimine (10, 20 mg/kg), whereas females were tested with three doses (10, 20, 30 mg/kg). The data for open zone occupancy, open zone entries, and total distance traveled in the elevated zero maze test are shown in Fig. 4.

Two-way ANOVAs using the 0, 10, and 20 mg/kg norbinaltorphimine doses were used to analyze the effects of norbinaltorphimine and test for sex × norbinaltorphimine pretreatment interactions. The two-way ANOVA indicated a main effect of norbinaltorphimine in oxytocin-treated mice for open zone occupancy (norbinaltorphimine<vehicle; *F*_2,59_ = 4.350, *p* = 0.0173), entries (*F*_2,59_ = 6.122, *p* = 0.0038), and total distance traveled (*F*_2,59_ = 4.872, *p* = 0.0110) but no main effect of sex (occupancy: *F*_1,59_ = 0.3830, *p* = 0.5384; entries: *F*_1,59_ = 0.7636, *p* = 0.3858; distance: *F*_1,59_ = 1.349, *p* = 0.2501). A norbinaltorphimine × sex interaction effect was observed for open zone entries (*F*_2,59_ = 3.158, *p* = 0.0498) and total distance traveled (*F*_2,59_ = 3.906, *p* = 0.0255), but not for open zone occupancy (*F*_2,59_ = 1.664, *p* = 0.1981). *Post hoc* comparisons of the main norbinaltorphimine effect (i.e., regardless of sex) demonstrated that 20 mg/kg norbinaltorphimine (*p* = 0.0132), but not 10 mg/kg norbinaltorphimine (*p* = 0.5152) inhibited the effect of oxytocin in the open zone occupancy. However, *post hoc* comparisons did not indicate an effect of 10 mg/kg (entries: *p* = 0.1586; distance: *p* = 0.2221) or 20 mg/kg norbinaltorphimine (entries: *p* = 0.1586; distance: *p* = 0.2221) on the open zone entries or total distance traveled. *Post hoc* tests that were conducted separately for the male and female data to follow up the norbinaltorphimine × sex interaction indicated that 20 mg/kg norbinaltorphimine (entries: *p* = 0.0317; distance: *p* = 0.0163), but not 10 mg/kg (entries: *p* = 0.2083; distance: *p* = 0.4638), decreased open zone entries and total distance traveled in males, whereas neither 10 mg/kg norbinaltorphimine (entries: *p* = 0.3951; distance: *p* = 0.2523) nor 20 mg/kg norbinaltorphimine (entries: *p* = 0.7976; distance: *p* = 0.4437) had an effect on total distance traveled in females. Note that a higher dose of norbinaltorphimine (30 mg/kg) was tested in females and appeared to have effects similar to 20 mg/kg in males.

There was a main effect of oxytocin treatment on open zone occupancy for vehicle-pretreated mice (oxytocin > vehicle; *F*_1,37_ = 23.41, *p* < 0.0001), but no effect of sex (*F*_1,37_ = 1.361, *p* = 0.2508) and no oxytocin × sex interaction (*F*_1,37_ = 1.511, *p* = 0.2267). For measures of open zone entries and total distance traveled, a main effect of oxytocin (oxytocin > vehicle; entries: *F*_1,37_ = 22.15, *p* < 0.0001; distance: *F*_1,37_ = 23.62, *p* < 0.0001) and an oxytocin × sex interaction (entries: *F*_1,37_ = 7.153, *p* = 0.0111; distance: *F*_1,37_ = 4.694, *p* = 0.0368) but no main effect of sex (entries: *F*_1,37_ = 0.2335, *p* = 0.6318; distance: *F*_1,37_ = 1.399, *p* = 0.2444), were observed. *Post hoc* tests indicated that oxytocin increased open zone entries in males (*p* < 0.0001) but not in females (*p* = 0.1110) and total distance traveled in males (*p* = 0.0001) and in females (*p* = 0.0369).

There was no main effect of norbinaltorphimine pretreatment alone (occupancy: *F*_1,32_ = 2.032, *p* = 0.1637; entries: *F*_1,32_ = 2.947, *p* = 0.0957; distance: *F*_1,32_ = 0.2622, *p* = 0.6122) and no norbinaltorphimine × sex interaction (occupancy: *F*_1,32_ = 0.4665, *p* = 0.4995; entries: *F*_1,32_ = 1.156, *p* = 0.2904; distance: *F*_1,32_ = 1.946, *p* = 0.1727) in vehicle-treated mice. However, a main effect of sex (males < females; occupancy: *F*_1,32_ = 4.304, *p* = 0.0462; entries: *F*_1,32_ = 6.811, *p* = 0.0137; distance: *F*_1,32_ = 9.940, *p* = 0.0035) was demonstrated for each measure reported.

These data suggest that blockade of κ−opioid receptors has an opposite effect of μ−opioid receptor blockade on the anxiolytic-like effects of oxytocin in male mice.

### Effect of opioid receptor antagonism on the antidepressant-like effect of oxytocin in male and female mice using the tail suspension test

**Fig. 5:**
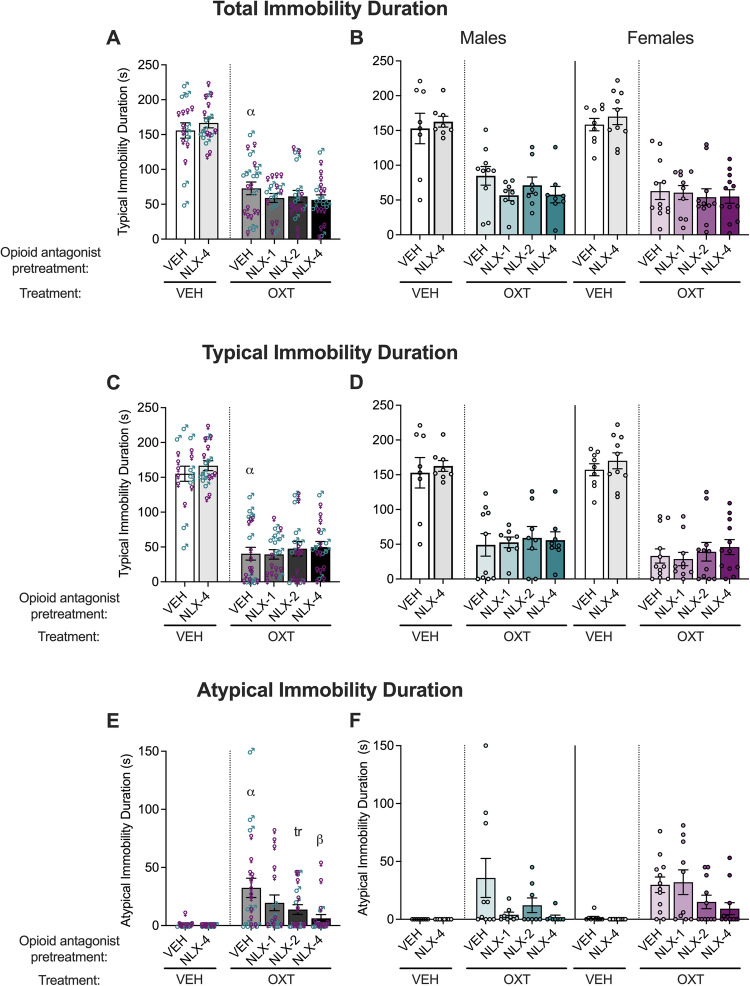
Naloxone blocks oxytocin-induced atypical immobility duration but not oxytocin-induced antidepressant-like (typical immobility) behavior. Six experimental groups were tested as VEH + VEH (*n*, males = 8, females = 9), NLX-4 + OXT (*n*, males = 8, females = 10), VEH + OXT (*n*, males = 10, females = 12), NLX-1 + OXT (*n*, males = 8, females = 10), NLX-2 + OXT (*n*, males = 8, females = 11), and NLX-4 + OXT (*n*, males = 8, females = 12). Oxytocin treatment (500 ng/mouse OXT; intracerebroventricular infusion) reduced total (**A**) and typical (**C**) immobility duration and increased atypical (**E**) immobility duration in vehicle-pretreated mice. Data are presented as means ± standard errors of the means. Naloxone pretreatment (1–4 mg/kg NLX; subcutaneous injection) did not have an additional effect on the total (**A**) or typical (**C**) immobility duration in the tail suspension test in vehicle- or oxytocin-treated male and female mice. Separate male and female data for open zone occupancy (**B**), entries (**D)**, and total distance traveled (**F**) are also shown. However, naloxone decreased the oxytocin-induced atypical immobility duration (**E**). ^α^*p* < 0.05, difference from VEH + VEH; ^β^*p* < 0.05, ^tr^*p* = 0.05, difference.

We also tested the hypothesis that opioid receptor blockade would affect the antidepressant-like effect of oxytocin. We administered vehicle or naloxone subcutaneously 5 min prior to intracerebroventricular administration of vehicle or oxytocin. The data for total, typical, and atypical immobility duration in the tail suspension test are shown in Fig. 5.

A main effect of oxytocin treatment was demonstrated by comparing vehicle *vs*. oxytocin in vehicle-pretreated mice such that oxytocin decreased total (*F*_1,35_ = 32.96, *p* < 0.0001) and typical (*F*_1,35_ = 62.52, *p* < 0.0001) immobility duration and increased atypical immobility duration (*F*_1,35_ = 10.85, *p* = 0.0023). There was no main effect of sex (total: *F*_1,35_ = 0.3308, *p* = 0.5689; typical: *F*_1,35_ = 0.1672, *p* = 0.6851; atypical: *F*_1,35_ = 0.05623, *p* = 0.8139) and no oxytocin × sex interaction (total: *F*_1,35_ = 0.9328, *p* = 0.3408; typical: *F*_1,35_ = 0.4942, *p* = 0.4867; atypical: *F*_1,35_ = 0.1400, *p* = 0.7106).

No main effect of naloxone pretreatment was observed in oxytocin-treated mice on total (*F*_3, 71_ = 0.9545, *p* = 0.4191) or typical (*F*_3, 71_ = 0.3614, *p* = 0.7811) immobility duration. However, we observed a main effect of naloxone pretreatment on atypical immobility duration in oxytocin-treated mice (naloxone < vehicle; *F*_3, 71_ = 3.701, *p* = 0.0155). We did not observe a main effect of sex (total: *F*_1, 71_ = 1.325, *p* = 0.2536; typical: *F*_1, 71_ = 3.900, *p* = 0.0522; atypical: *F*_1, 71_ = 1.712, *p* = 0.1949) or naloxone × sex interaction (total: *F*_3, 71_ = 0.5508, *p* = 0.6493; typical: *F*_3, 71_ = 0.1111, *p* = 0.9533; atypical: *F*_3, 71_ = 1.352, *p* = 0.2646) for either of the reported measures. *Post hoc* tests of the combined male and female data demonstrated that the 4 mg/kg dose of naloxone increased atypical immobility duration (*p* = 0.0058), whereas the 1 mg/kg (*p* = 0.0920) and 2 mg/kg (*p* = 0.0546) doses did not increase atypical immobility duration but demonstrated a trend toward statistical significance.

The vehicle *vs*. 4 mg/kg naloxone comparison in vehicle-treated mice showed no main effect of naloxone pretreatment (total: *F*_1,31_ = 0.6412, *p* = 0.4294; typical: *F*_1,31_ = 0.7300, *p* = 0.3994; atypical: *F*_1,31_ = 2.447, *p* = 0.1279) or sex (total: *F*_1,31_ = 0.2367, *p* = 0.6300; typical: *F*_1,31_ = 0.1920, *p* = 0.6643; atypical: *F*_1,31_ = 2.447, *p* = 0.1279) and no naloxone × sex interaction (total: *F*_1,31_ = 0.004605, *p* = 0.9463; typical: *F*_1,31_ = 0.01403, *p* = 0.9065; atypical: *F*_1,31_ = 2.447, *p* = 0.1279).

These data suggest that μ−opioid receptor blockade could potentiate the anxiolytic- but not the antidepressant-like effect of oxytocin, suggesting that these behaviors are mediated by different mechanisms. That naloxone could block oxytocin-induced atypical immobility duration further supports this dissociation and suggests that oxytocin may recruit an opioidergic mechanism or that oxytocin may share a downstream mechanism with the μ−opioid receptor.

## Discussion

Consistent with previous data from our laboratory [12] and others [4, 47, 48], we demonstrated that intracerebroventricular oxytocin administration reduced measures of anxiety- and depression-like behavior in male and female mice. We then showed that μ−opioid receptor blockade potentiated the anxiolytic-like effect of oxytocin, whereas κ−opioid receptor blockade, inhibited the anxiolytic-like effect of oxytocin. μ−Opioid receptor blockade with naloxone had no effect on depression-like behavior (typical immobility duration in the tail suspension test) per se. However, naloxone blocked an atypical immobility curling response that is suggested to be opioid-mediated [34]. Altogether, the present study shows a functional interaction between the opioid and oxytocin systems in anxiety- and depression-like behaviors.

The effects of naloxone in the present study are consistent with reports of naloxone potentiating the effect of FDA-approved anxiolytic drugs and γ-aminobutyric acid (GABA) receptor agonists [49–56], which suggests a consistent role of μ−opioid receptor antagonists in modulating anxiolytic-like behavior. For example, when coadministered with subeffective doses of benzodiazepines, i.e., positive allosteric modulators at the GABA receptor, naloxone (10 mg/kg intraperitoneally) potentiated their anxiolytic-like effect in the elevated plus maze [50–55]. Note, however, that others demonstrated an attenuation of the anxiolytic-like effects of benzodiazepines with naloxone in rodents [56–58]. Further, in the present study, CTAP, a selective μ−opioid receptor antagonist, administered intracerebroventricularly potentiated the anxiolytic-like effect of oxytocin, demonstrating that the anxiolytic-like effect of oxytocin is potentiated by blocking the central action of endogenous opioids at μ−opioid receptors.

Using maternal and social behavior models, studies involving opioid receptor agonists and antagonists have demonstrated a similar profile of opioid system interactions [59–67]. For example, in late pregnancy in rats (i.e., 1-2 days before gestation), naloxone (5 mg/kg; subcutaneous) increased plasma oxytocin concentrations by disinhibiting its release from the neurohypophysis [59–61], whereas morphine, a μ−opioid receptor agonist, reduced plasma oxytocin levels [62]. These results support an inhibitory role of endogenous opioids on the oxytocin system in the hypothalamus. A recent report on male and female rhesus macaques demonstrated that naloxone (0.5-2 mg; intranasal) enhanced measures of social attention and cognition when coadministered with oxytocin (24 IU, intranasal) [66]. Additionally, a case study of a 13-year-old boy showed that naltrexone (100 mg/day; oral) and oxytocin (6 IU every 1-3 days; intranasal) combination therapy improved hypothalamic-associated obesity [67]. Altogether, these studies support the hypothesis that μ−opioid receptor antagonism may enhance the efficacy of oxytocin on multiple biological measures.

We also investigated whether blockade of κ−opioid receptors had similar or different effects on the anxiolytic-like effects of oxytocin. κ−opioid receptors are highly expressed in emotion-mediating brain structures, and naloxone and CTAP can bind to κ−opioid receptors, albeit with significantly reduced affinity and potency compared to their binding affinity to μ−opioid receptors. In the present study, we observed that norbinaltorphimine inhibited the anxiolytic-like effect of oxytocin. This inhibitory effect of norbinaltorphimine supports the well-established hypothesis that κ−opioid receptors mediate effects opposite of μ-opioid receptors, such as dysphoria and dysphoric-like behavior [43, 68–71].

These results are consistent with our previous report showing that oxytocin reduces anxiety-like behavior in both males and females, with a more pronounced effect in males [12]. We also demonstrated in the previous study that oxytocin was more effective in females in proestrus/estrus compared with females in metestrus/estrus and in ovariectomized females that were supplemented or not with estrogen and progesterone. As such, follow-up studies could investigate whether opioid receptor antagonism would have similar effects in females with various hormonal status. Note that in the present study, µ-opioid receptor antagonists potentiated the effect of oxytocin in both sexes. This may suggest a sex difference in the regulation of oxytocin and/or oxytocin receptor-expressing neurons in male and female mice by opioids and has implications for possible future clinical use.

As demonstrated in our previous study, oxytocin reduced measures of depression-like behavior (i.e., total and typical immobility duration**)**. We also reported that oxytocin increased the atypical immobility response, an additional measure that captures the amount of time that mice spend immobile while the trunk is curled. This atypical immobility behavior phenotype is noteworthy as this behavior is not common in drug-naïve mice. In an early report [27], naloxone (0.5-2 mg/kg, intraperitoneally) blocked curling behavior without affecting typical immobility duration, indicative of an opioid receptor-mediated mechanism. This report also demonstrated that antidepressants that inhibit norepinephrine and/or serotonin reuptake (e.g., imipramine, venlafaxine, duloxetine, desipramine, and citalopram) did not affect atypical immobility, a sub-phenotype of curling, suggesting that the opioids have different or additional mechanisms than typical selective norepinephrine and/or serotonin reuptake inhibitors [27]. Consistent with our results, opioids selectively increased atypical mobility and immobility (curling), while decreasing typical immobility duration, regardless of whether they inhibited monoamine reuptake (levorphanol, -methadone) or not (morphine). Likewise, other studies replicated this effect by showing that some plant extracts that have antidepressant-like activity can decrease typical immobility while increasing atypical mobility and immobility duration [72–76].

As such, the present findings showing a blockade of oxytocin-induced curling with naloxone further support the hypothesis that like opioids, oxytocin recruits a different mechanism to produce atypical immobility in the tail suspension test. A survey of the literature suggests that the atypical behavior phenotype may be mediated by downstream activation of mitogen-activated protein kinase (MAPK). First, both opioids [77–79] and oxytocin [80, 81] acutely activate the MAPK pathway. Second, acute inflammation, which also triggers MAPK activation [82, 83], is distinguished by curling (atypical mobility and immobility) in mice [84, 85]. Finally, naloxone inhibits MAPK activation, which may explain its inhibition of atypical immobility [86]. This potential role for MAPK activation should be further explored; this may give further insight into the cellular mechanism of oxytocin and opioid receptors.

Multiple mechanisms can be hypothesized to explain the interaction of naloxone, CTAP, and norbinaltorphimine with oxytocin on anxiety-like behavior in the elevated zero maze. First, we hypothesize that naloxone and CTAP increase the release of oxytocin and the activity of oxytocin neurons in the hypothalamus, as shown in earlier studies [87–90]. These studies established that μ−opioid receptors have regulatory control over oxytocin receptors in the paraventricular and supraoptic nuclei of the hypothalamus. Second, we hypothesize that naloxone and CTAP can directly or indirectly increase the responsiveness of oxytocin receptor-expressing neurons in the extended amygdala, whereas norbinaltorphimine can decrease the responsiveness of these neurons. Previous studies have demonstrated that oxytocin, μ−, and κ−opioid receptors are expressed on GABAergic neurons in the amygdala [18, 91] and predominantly couple inhibitory G-proteins [92, 93]. Others have demonstrated that κ−opioid receptors may bind to stimulatory G-proteins [92] and that oxytocin receptors predominantly couple stimulatory G-proteins [94, 95]. Altogether, these data suggest that μ−opioid receptor antagonists, such as naloxone and CTAP, may disinhibit GABAergic neurons allowing for increased responsiveness to oxytocin in the amygdala. κ−Opioid receptor antagonists may inhibit GABAergic neurons and thus decrease responsiveness to oxytocin in this region. As in the hypothalamus, this suggests a regulatory control of amygdala neurons.

Such intriguing interactions between opioid receptor antagonists and oxytocin outlined in our study may be of clinical relevance given that naltrexone is an FDA-approved medication for the treatment of alcohol use disorders [96], which presents high comorbidity with mood and anxiety disorders, and oxytocin is currently under study for the same indication (ClinicalTrials.gov: NCT03878316). This is particularly important given the need for new pharmacotherapeutics for the treatment of psychiatric disorders, including substance use disorders [97]. Of interest would be clinical trials that are designed to investigate the combined effects of oxytocin and naltrexone [98].

In conclusion, we provide novel evidence for an interaction between the endogenous opioid and oxytocin systems in both male and female mice. Our data demonstrate that the blockade of μ−opioid receptors potentiated the anxiolytic-like effect of oxytocin, whereas the blockade of κ−opioid receptors inhibited the anxiolytic-like effect of oxytocin, suggesting that the μ− and κ−opioid receptor systems differentially modulate the function of oxytocin with respect to anxiety-like behavior. In addition, we demonstrate a key difference in pharmacological mechanisms underlying our behavioral tests such that naloxone potentiated the effect of oxytocin on anxiety-like behavior but not on depression-like behavior. Altogether, this study highlights the importance of the opioid and oxytocin interaction for emotion regulation and supports differences in neural mechanisms underlying depression and anxiety.
